# Early vs. persistent microvascular obstruction following primary PCI- two pathologies or one? A cardiovascular magnetic resonance study

**DOI:** 10.1186/1532-429X-16-S1-P192

**Published:** 2014-01-16

**Authors:** Ananth Kidambi, David P Ripley, Akhlaque Uddin, Adam K McDiarmid, Peter P Swoboda, Tarique A Musa, Gavin Bainbridge, John P Greenwood, Sven Plein

**Affiliations:** 1 Cardiology, Multidisciplinary Cardiovascular Research Centre & Leeds Institute of Genetics, Health and Therapeutics, Leeds, UK

## Background

20-40% of reperfused ST-elevation acute myocardial infarction (STEMI) features microvascular obstruction (MO), which confers adverse prognosis. Different CMR sequences are commonly used to detect MO: first pass perfusion (FPP), early gadolinium enhancement (EGE), and late gadolinium enhancement (LGE). FPP and EGE are more sensitive than LGE for the detection of MO. However, only MO by LGE has been shown to confer prognostic information. It is unclear whether these three methods detect separate pathologies, or whether differences in MO appearances merely reflect contrast distribution over time. We aimed to determine how appearances between these methods are related.

## Methods

60 patients underwent CMR at 3.0T within 3 days following reperfused first STEMI. MO imaging was performed at identically-planned basal, mid-ventricular and apical short-axis slices. FPP imaging was performed during administration of 0.1 mmol/kg Gd-DTPA contrast. 4 minutes after contrast administration, EGE imaging was performed, followed by LGE imaging at both 10 minutes and at 20 minutes. MO was identified as a dark core within infarcted myocardium. We compared area and transmural extent of MO for each method on a per-patient and a per-slice basis.

## Results

**Figure 1:**
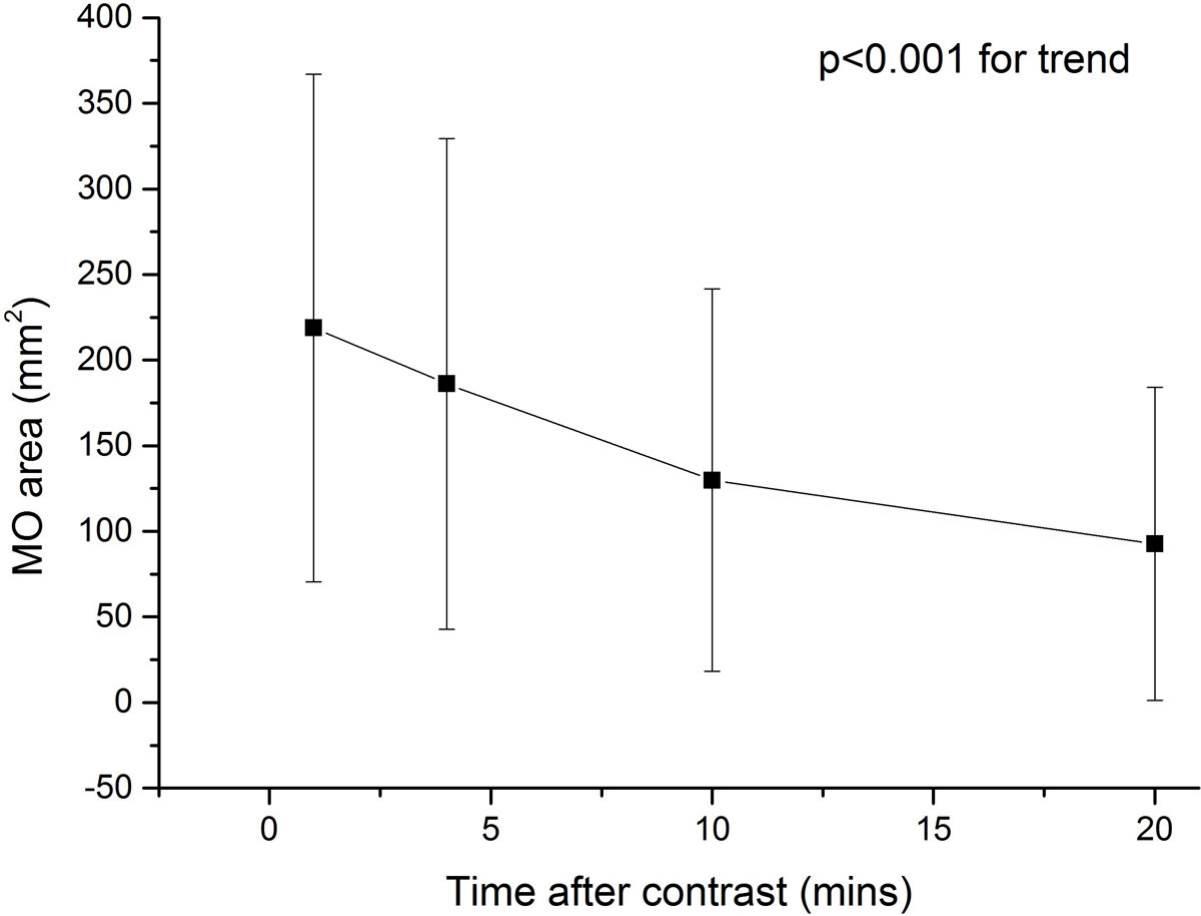
**Decrease in apparent area of MO visible after administration of contrast**.

**Figure 2:**
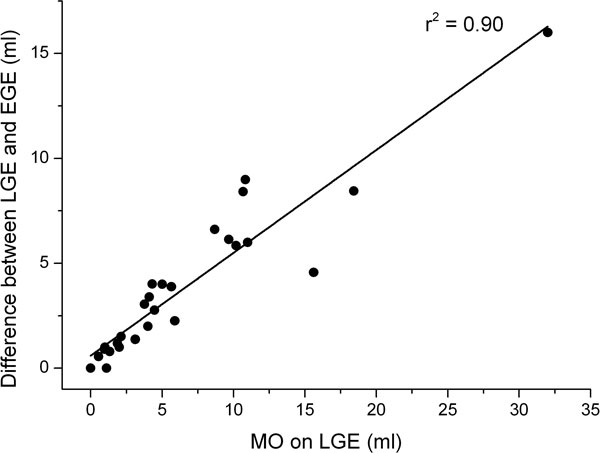
**Change in MO volume over time is closely correlated with volume of MO**.

29 patients (48%) had MO. All patients with MO on LGE also had MO on FPP or EGE, whereas LGE at 10 minutes failed to detect MO in 9 patients (31%) with MO on FPP, and 8 patients (28%) on EGE. Of 13 patients with MO volume <5 ml on FPP, 12 (92%) had no MO visible on LGE at 20 minutes. Average visible area of MO per slice decreased with time of measurement (p < 0.001 for trend, Figure 1). MO area by FPP and EGE correlated with LGE at 20 minutes (r = 0.80; p < 0.001 and r = 0.80; p < 0.001) but MO volume (per patient) by FPP and EGE was on average 236% and 200% larger than LGE. Decrease in MO volume over time correlated strongly with size of MO (r = 0.95, p < 0.01, Figure 2) and transmural extent of MO (r = 0.77, p < 0.01).

## Conclusions

The reduction in visible size of MO is proportional to extent of MO and time from contrast administration. Contrast diffusion into the MO zone may be responsible for reduction in MO size between the methods rather than differing clinical or imaging factors. Smaller areas of MO on EGE and FPP become undetectable on LGE, while larger areas are detectable but smaller on LGE. MO by LGE therefore identifies more extensive MO than FPP or EGE, possibly explaining its higher predictive value.

## Funding

JPG and SP receive a research grant from Philips Healthcare. SP is funded by British Heart Foundation fellowship (FS/10/62/28409).

